# A multicenter prospective phase II study of first-line modified FOLFIRINOX for unresectable advanced pancreatic cancer

**DOI:** 10.18632/oncotarget.22795

**Published:** 2017-11-30

**Authors:** Kensaku Yoshida, Takuji Iwashita, Shinya Uemura, Akinori Maruta, Mitsuru Okuno, Nobuhiro Ando, Keisuke Iwata, Junji Kawaguchi, Tsuyoshi Mukai, Masahito Shimizu

**Affiliations:** ^1^ First Department of Internal Medicine, Gifu University Hospital, Gifu, Japan; ^2^ Department of Gastroenterology, Gifu Prefectural General Medical Center, Gifu, Japan; ^3^ Department of Gastroenterology, Gifu Municipal Hospital, Gifu, Japan

**Keywords:** adverse events, febrile neutropenia, dose modification, biliary drainage, risk factor

## Abstract

**Background:**

FOLFIRINOX (FX) has been reported as an effective treatment for unresectable advanced pancreatic cancer. However, FX is associated with a high incidence of adverse events (AEs). A previous phase II study in Japan showed high incidences of hematological AEs, including febrile neutropenia (22.2%). A modified FX regimen (mFX) may decrease the rates of AEs and be more effective than FX by improving the treatment compliance.

**Aims:**

To assess the safety and efficacy of first-line mFX for unresectable advanced pancreatic cancer.

**Patients and methods:**

This was as a multicenter prospective phase II study in chemotherapy-naïve Japanese patients with pathologically confirmed unresectable advanced pancreatic adenocarcinoma or adenosquamous carcinoma. Treatment with mFX (85 mg/m2 oxaliplatin, 150 mg/m2 irinotecan, and 200 mg/m2 l-leucovorin, followed by 46-h continuous infusion of 2400 mg/m2 5-fluorouracil) was administered every 2 weeks. The primary endpoint was the response rate. The secondary endpoints were overall survival, progression-free survival, and safety.

**Results:**

Thirty-one patients (18 men; median age, 64 years) were enrolled. A median of 13 treatment cycles were administered during a median follow-up period of 14.2 months. The response rate, median overall survival, and median progression-free survival were 38.7%, 14.9 months, and 7.0 months, respectively. Grade 3 or 4 AEs included neutropenia (83.9%), febrile neutropenia (16.1%), peripheral sensory neuropathy (9.7%), thrombocytopenia (6.5%), diarrhea (6.5%), anorexia (6.5%), and vomiting (3.2%).

**Conclusion:**

Compared to FX, mFX may result in fewer Grade 3 or 4 non-hematological AEs, with a comparable response rate. However, further efforts might be required to reduce hematological AEs.

## INTRODUCTION

Pancreatic cancer is the fourth leading cause of cancer-related death in Japan, with 31,866 deaths reported annually [1]. The 5-year survival rate of pancreatic cancer is reported as only 8.3%, owing to the fact that pancreatic cancer is typically found at an advanced stage [2]. At the time of diagnosis, almost 50% of patients have metastatic lesions and 30% are diagnosed with locally advanced pancreatic cancer [3]. Given the above-mentioned situation, the development of effective and tolerable chemotherapy is highly important for the management of advanced pancreatic cancer.

In 1997, a study comparing gemcitabine (GEM) and 5-fluorouracil (5-FU) for advanced pancreatic cancer showed an improvement in overall survival (OS) with GEM [4]. Since then, GEM has been a key chemotherapy agent for pancreatic cancer. In 2011, a French phase II/III study by Conroy et al. comparing FOLFIRINOX (FX; a combination of 5-FU, oxaliplatin, irinotecan, and leucovorin) versus GEM in metastatic pancreatic cancer showed significant improvements in the median OS (11.1 vs. 6.8 months, p<0.001) and progression-free survival (PFS; 6.4 vs. 3.3 months, p<0.001), as well as an increased response rate (RR; 31.6% vs. 9.4%) [5]. Following this study, a phase II study of FX for metastatic pancreatic cancer in a Japanese cohort was conducted and showed equivalent efficacy as in the original report [6]. However, in terms of its safety profile, the phase II/III study reported that FX was associated with significantly higher rates of grade 3 or 4 neutropenia (45.7% vs. 21.0%), diarrhea (12.7% vs. 1.8%), and sensory neuropathy (9.0% vs. 0%) compared to GEM [5]. The rates of toxicity in the Japanese phase II study of FX was also higher, especially for hematological toxicities such as grade 3 or 4 neutropenia (77.8%) and febrile neutropenia (FN; 22.2%) [6]. These studies reported that the higher toxicity was associated with higher rates of subsequent dose reductions and treatment delays.

Based on these previous two studies, FX can be concluded to represent an effective treatment in patients with metastatic pancreatic cancer; however, its tolerability remains a concern. An initial dose reduction of FX might reduce the toxicity without compromising the efficacy by improving the compliance to the chemotherapy. Thus, we conducted this multicenter prospective phase II study of first-line modified FX (mFX) for unresectable advanced pancreatic cancer in a Japanese cohort.

## RESULTS

### Patient characteristics

Between October 2014 and March 2016, 31 patients were recruited and enrolled in this study from three tertiary care centers: Gifu University Hospital, Gifu Municipal Hospital, and Gifu Prefectural General Medical Center. The basic characteristics of the study patients are shown in Table 1. Treatment was discontinued in 29 patients (93.5%), owing to progression of primary disease in 24 patients (77.4%), adverse events in 4 patients (12.9%; neutropenia in 2, vomiting in 1 and peripheral sensory neuropathy in 1) and after R0 operation in 1 patient (3.2%).

**Table 1:** Baseline characteristics of patients with unresectable advanced pancreatic cancer treated with modified FOLFIRINOX

Age, year-old, median (Range)		64 (49-72)
Sex, n (%)	male	18 (58.1%)
	female	13 (41.9%)
ECOG PS, n (%)	0	25 (80.6%)
	1	6 (19.4%)
CA19-9, μ/mL	median (range)	787.3 (2-24469.2)
Pathological type, n (%)	adenocarcinoma	30 (96.8%)
	adenosquamous carcinoma	1 (3.2%)
Disease extent, n (%)	locally advanced	10 (32.3%)
	metastatic	21 (67.7%)
Site of primary tumor, n (%)	head	15 (48.4%)
	body/tail	16 (51.6%)
Site of metastasis, n (%)	liver	13 (41.9%)
	lung	3 (9.7%)
	bone	3 (9.7%)
	others	3 (9.7%)
Biliary drainage, n (%)	no	23 (74.2%)
	yes	8 (25.8%)
UGT1A1(^*^6/^*^28), n (%)	Wild/Wild	21 (67.7%)
	Wild/heterozygous	5 (16.1%)
	Heterozygous/wild	5 (16.1%)

ECOG PS, Eastern Cooperative Oncology Group Performance Status; UGT1A1, uridine diphosphate-glucuronosyltransferase 1A1

### Efficacy of the modified FOLFIRINOX

**Figure 1:**
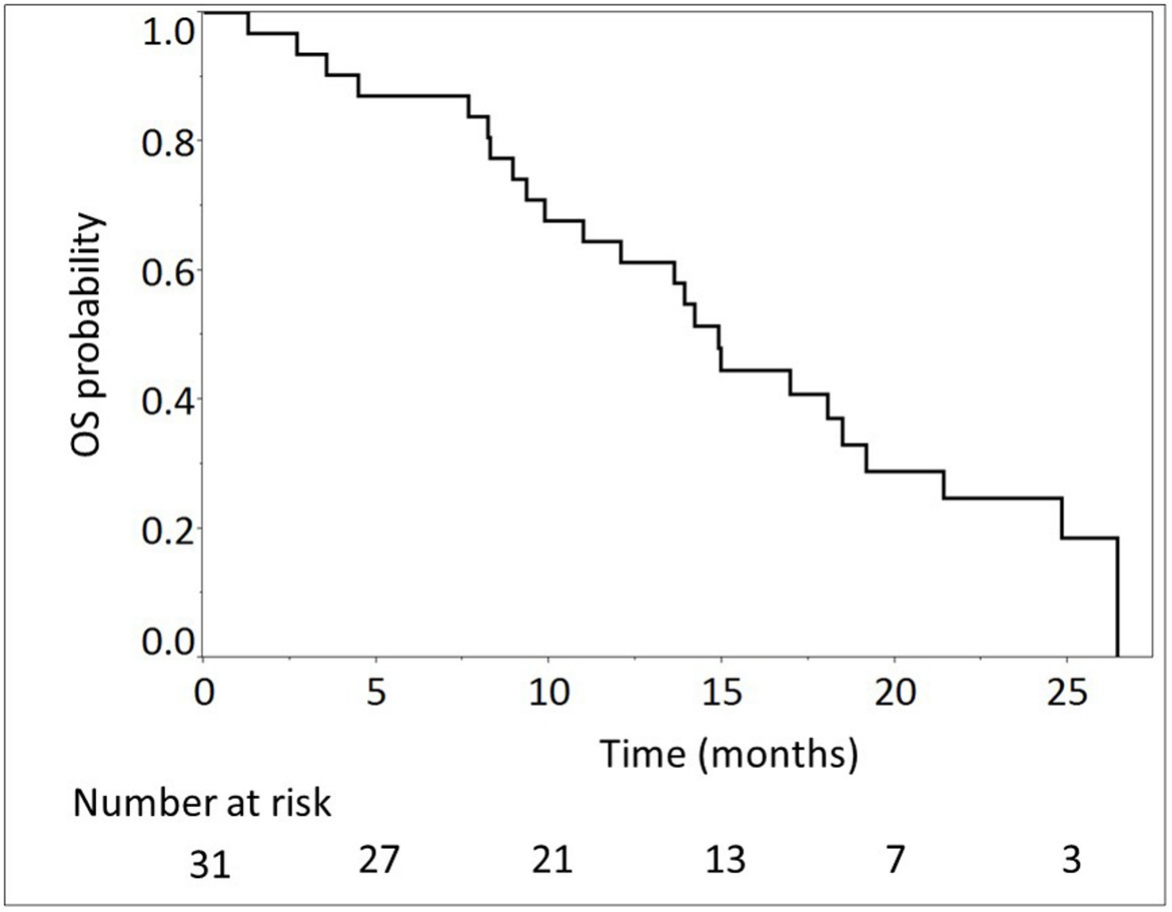
Overall survival (OS) of 31 patients with unresectable advanced pancreatic cancer treated with modified FOLFIRINOX The median OS was 14.9 months (95% confidence interval, 9.9-19.2).

**Figure 2:**
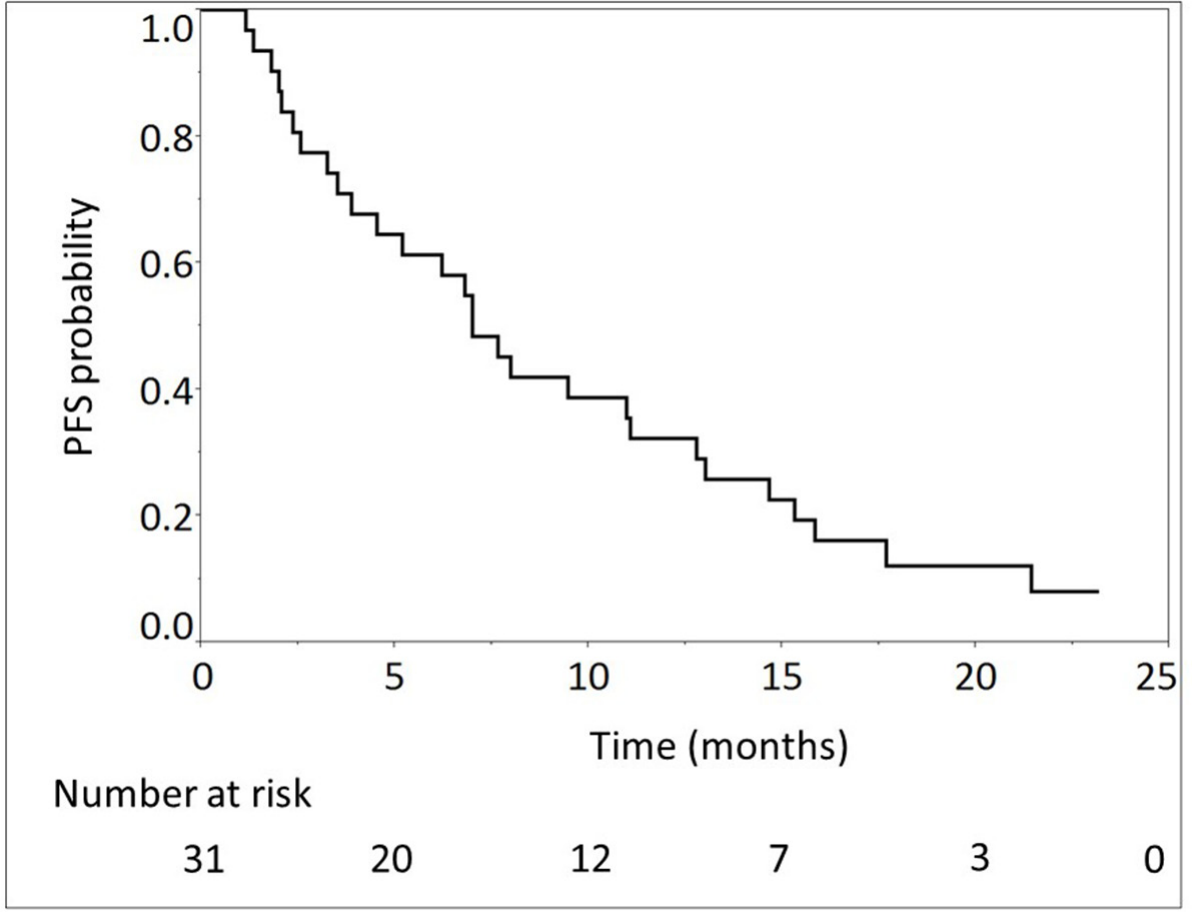
Progression-free survival (PFS) of 31 patients with unresectable advanced pancreatic cancer treated with modified FOLFIRINOX The median PFS was 7.0 months (95% confidence interval, 3.9-11.1).

**Figure 3:**
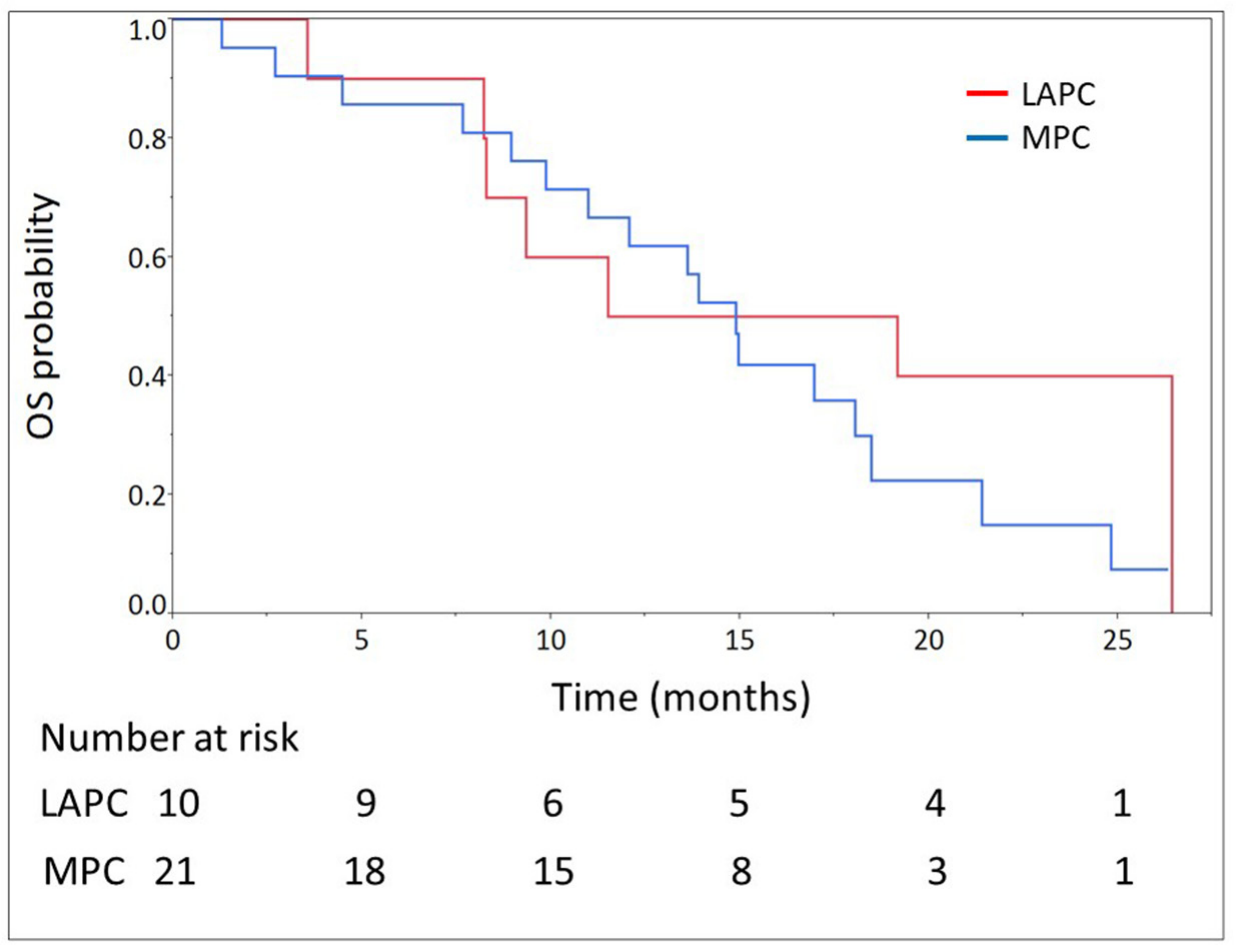
Overall survival (OS) of 21 patients with metastatic pancreatic cancer (MPC) and 10 patients with locally advanced pancreatic cancer (LAPC) treated with modified FOLFIRINOX The median OS were 14.9 months (95% confidence interval, 9.9-18.5) and 16.7 months (95% confidence interval, 3.6-26.4) in patients with MPC and LAPC, respectively.

**Figure 4:**
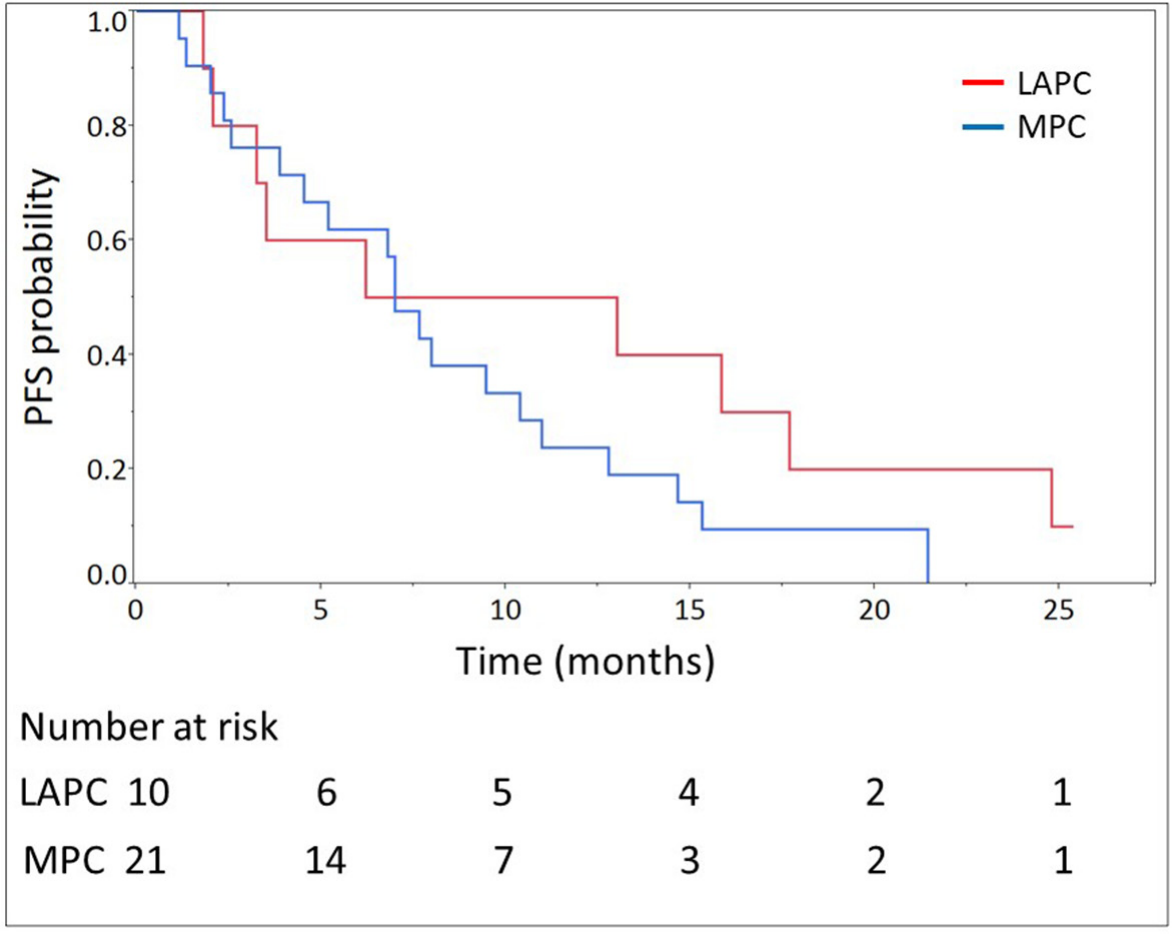
Progression-free survival (PFS) of 21 patients with metastatic pancreatic cancer (MPC) and 10 patients with locally advanced pancreatic cancer (LAPC) treated with modified FOLFIRINOX The median PFS were 7.0 months (95% confidence interval, 3.9-11.0) and 9.6 (95% confidence interval, 1.8-17.7) in patients with MPC and LAPC, respectively.

The median number of cycles administered was 13 (range, 2-45) in all patients. The median relative dose intensities were as follows: oxaliplatin, 59.6%; irinotecan, 72.8%; 5-FU, 77.9%; and l-leucovorin, 82.5%. A total of 28 (90.3%) and 29 (93.5%) patients required dose reductions and dose delays, respectively, mainly due to neutropenia (Table 2). The median follow-up time was 14.2 months. At the time of analysis, 24 (77.4%) deaths had occurred. Of the 31 evaluable patients, response evaluation showed a partial response in 12 patients (38.7%), stable disease in 11 patients (35.5%), and progressive disease in 8 patients (25.8%). The RR was 38.7% (95% CI, 23.7-56.2) and the disease control rate was 74.2% (95% CI, 56.8-86.3). The estimated 1-year OS rate was 64.5% (95% CI, 46.6-79.1), with a median OS of 14.9 months (95% CI, 9.9-19.2; Figure 1). The estimated 1-year PFS rate was 32.3% (95% CI, 18.3-50.3), with a median PFS of 7.0 months (95% CI, 3.88-11.1; Figure 2, Table 3). The RR, median OS, and median PFS in patients with locally advanced and metastatic pancreatic cancer were 50% (5/10; 95% CI, 23.7-76.3) and 33,3% (7/21; 95% CI, 17.2-54.6), 16.7 months (95% CI, 3.6-26.4) and 14.9 months (95% CI, 9.9-18.5) (Figure 3), and 9.6 months (95% CI, 1.8-17.7) and 7.0 months (95% CI, 3.9-11.0) (Figure 4), respectively.

**Table 2:** Treatment duration and drug delivery of patients with unresectable advanced pancreatic cancer treated with modified FOLFIRINOX

Median cycles of treatment, number (range)	13(2-37)
Median relative dose intensity, % (range)	
Oxaliplatin	59.59% (13.8-100%)
Irinotecan	72.79% (43.9-100%)
5-FU	77.92% (32.1-100%)
*l*-Leucovorin	82.5% (48.0-100%)
Dose reduction (total patients), n (%)	
Overall	28 (90.3%)
Oxaliplatin	28 (90.3%)
Irinotecan	24 (77.4%)
5-FU	5 (16.1%)
Delayed cycles, n (%)	
Overall	29 (93.5%)

**Table 3:** Efficacy results of patients with unresectable advanced pancreatic cancer treated with modified FOLFIRINOX

Objective response rate	
Complete Response, n (%)	0 (0%)
Partial Response, n (%)	12 (38.7%)
Stable Disease, n (%)	11 (35.5%)
Progressive Disease, n (%)	8 (25.8%)
Response rate, n (%, 95% CI)	12 (38.7%, 23.7-56.2)
Disease control rate, n (%, 95% CI)	23 (74.2%, 56.8-86.3)
1-year overall survival rate, % (95% CI)	64.5 (46.6-79.1)
Overall survival, months, median (95% CI)	14.9 (9.9-19.2)
1-year progression-free survival rate, % (95% CI)	32.3 (18.3-50.3%)
Progression-free survival, months, median (95% CI)	7.0 (3.9-11.1)

95% CI, 95% confidence interval

### Second-line chemotherapy and surgical resection

Of the 29 patients who discontinued mFX, 25 patients received second-line chemotherapy with GEM plus nab-paclitaxel (n=19), GEM (n=2), or S-1 (n=4), while 4 patients did not receive any second-line chemotherapy. Two patients proceeded to undergo surgery after treatment with 5 and 13 cycles of mFX, respectively. One patient had R1 resection and the other patient had R0 resection on the final pathological evaluation. Both patients are still alive. One patient is continuing mFX and the other patient is receiving S-1 as adjuvant chemotherapy.

### Safety profile

Twenty-seven (87.1%) of the 31 patients experienced grade ≥3 adverse events. The most common grade ≥3 hematologic adverse events were neutropenia (83.9%), FN (16.1%), and thrombocytopenia (6.5%). G-CSF was administered in 18 patients (58.1%) due to grade ≥3 neutropenia. FN occurred only during the first cycle and in 50% (4/8) of patients with biliary drainage whereas the incidence of FN in patients without biliary drainage was 4.3% (1/23). The most common grade ≥3 non-hematologic adverse events were peripheral sensory neuropathy (9.7%), diarrhea (6.5%), anorexia (6.5%), and vomiting (3.2%) (Table 4). There were no treatment-related deaths. Four (12.9%) of the 31 patients discontinued the treatment due to the following adverse events: vomiting in 2 patients (6.5%), peripheral sensory neuropathy in 1 patient (3.2%), and neutropenia in 1 patient (3.2%). Biliary tract-related adverse events (cholangitis and cholecystitis in 2 patients each) were recognized in 4 patients (12.9%), including 3 patients who already had a biliary stent. Among them, cholangitis was managed with stent exchange and cholecystitis was managed with percutaneous transhepatic gallbladder drainage followed by laparoscopic cholecystectomy.

**Table 4:** Adverse events of patients treated with unresectable advanced pancreatic cancer with modified FOLFIRINOX

Adverse events	Total	≧Grade 3
	n (%)	n (%)
Hematological toxicities,		
Neutropenia	29 (93.5%)	26 (83.9%)
Febrile neutropenia	5 (16.1%)	5 (16.1%)
Thrombocytopenia	6 (19.4%)	2 (6.5%)
Anemia	14 (45.2%)	0 (0%)
Non-hematological toxicities		
Peripheral sensory neuropathy	18 (58.1%)	3 (9.7%)
Diarrhea	13 (41.9%)	2 (6.5%)
Anorexia	6 (19.4%)	2 (6.5%)
Vomiting	5 (16.1%)	1 (3.2%)
Nausea	4 (12.9%)	1 (3.2%)
Malaise	2 (6.5%)	0 (0%)
Mucositis oral	2 (6.5%)	0 (0%)
Erythema	1 (3.2%)	0 (0%)
Cholinergic syndrome	1 (3.2%)	0 (0%)
Interstitial pneumonia	1 (3.2%)	0 (0%)
Elevated AST	1 (3.2%)	0 (0%)

AST, aspartate aminotransferase

## DISCUSSION

This phase II open label study was carried out to investigate the efficacy and safety of the mFX regimen for unresectable advanced pancreatic cancer, including both locally advanced and metastatic cancers. Overall, the RR was 38.7%, with a median follow-up time of 14.2 months. The median OS and PFS were 14.9 and 7.0 months, respectively. Grade 3 or 4 adverse events included neutropenia (84%), FN (16.1%), peripheral sensory neuropathy (9.7%), thrombocytopenia (6.5%), diarrhea (6.5%), anorexia (6.5%), and vomiting (3.2%).

In our study, the mFX regimen was decided based on the result of a phase I study of infusional 5-FU, leucovorin, oxaliplatin, and irinotecan for advanced colorectal cancer in a Japanese cohort, which was designed to determine the maximum tolerated dose. That study showed that the recommended dose in Japanese patients was irinotecan at 150 mg/m^2^, oxaliplatin at 85 mg/m^2^ and 5-FU at 2,400 mg/m^2^ [7]. A few studies reporting the efficacy and tolerability by initial dose reduction of FX as first-line chemotherapy for advanced pancreatic cancer have also been published. Mahaseth et al. performed a retrospective analysis of mFX in which only bolus 5-FU was discontinued [8]. Stein et al. also performed a phase II study of mFX with a 25% reduction of irinotecan and bolus 5-FU (irinotecan intravenously at 135 mg/m^2^ and intravenous bolus 5-FU at 300 mg/m^2^) [9]. Although no clear rationale was described in the former study, in the latter, the modified regimen was decided based on the authors’ retrospective analysis of FX, which showed that the majority of patients received attenuated doses of bolus 5-FU and irinotecan. As a result, the authors suggested that up-front dose attenuation of bolus 5-FU and irinotecan improved the tolerability without compromising the efficacy of the treatment.

The RR of 38.7% (95% CI 23.7-56.2%) in our study was similar to those of the phase II/III study of FX in France and the phase II study of FX in Japan (31.6% and 38.9%, respectively) [5, 6]. The PFS of 7.0 months (95% CI, 3.9-11.1 months) and OS of 14.9 months (95% CI, 9.9-19.2 months) in our study were also comparable with those of the FX of the phase II/III study in France (6.4 and 11.1 months, respectively) and phase II study in Japan (5.6 and 10.7 months, respectively) [5, 6]. Considering these data, our modified regimen of FX maintained its efficacy even with the dose reduction, although a direct comparison between these studies could not be performed due to the different basic characteristics of the study subjects. The most important difference is that our study cohort included patients with both locally advanced and metastatic pancreatic cancer as unresectable advanced pancreatic cancer. However, even if the results only in the patients with metastasis one were reviewed, the RR of 33.3%, median OS of 14.9 months, and median PFS of 7.0 months were comparable. Thus, in other words, even in the subgroup analyses of locally advanced and metastatic cancers, our results were comparable with those of FX in the previous studies [5, 6]. As expected, the PFS and OS in locally advanced cases tended to be slightly better than those in metastatic cases in this study. Although there were no significant differences in the results between locally advanced and metastatic cancers, this might be due to the small number of patients in the sub-group analyses.

Regarding the toxicity profile in this study, the hematological adverse event rates were not suppressed compared to the rates of the French phase II/III and the Japanese phase II study of FX. The rate of grade 3 or 4 neutropenia was 83.9% (95% CI, 67.4-92.9 %); this was similar to in the phase II study in Japan (77.8 %) and higher than that of the phase II/III study in France (45.7 %) [5, 6]. FN occurred in 16.1% (95% CI, 7.1-32.6 %) of patients in our study, and this was also similar to the rate in the Japanese phase II study (22.2 %) and higher than that in the French phase II/III study (5.4 %) [5, 6]. Although FN occurred only in the first cycle and was successfully treated with administration of antibiotics and G-CSF, with a median recovery time of 3 days (range 2-6), careful observation during the first cycle is considered important to monitor for neutropenia and FN. Another point of view to reduce the rates of neutropenia and FN is prophylactic usage of G-CSF. The use of G-CSF was not permitted as prophylaxis in this study; however, 69.2 % of the patients were eventually treated with G-CSF due to grade 3 or 4 neutropenia or FN. Similar to in the phase II study of FX in Japan, which reported that severe infection and FN frequently occurred in patients with biliary stents at baseline [6], FN occurred in 50 % of patients with biliary stent placement for biliary obstruction in our study. These results suggest that careful observation of possible hematological adverse events from neutropenia or FN, especially in the first cycle and in patients with biliary stent placement, is required, even when the FX regimen is modified. Prophylactic administration of G-CSF, including pegylated filgrastim, might be one option to reduce the rates of serious adverse events. For other hematological adverse events, the rates of grade 3 or 4 thrombocytopenia and anemia in this study (6.5 % and 0 %) were lower than those in the FX phase II/III study in France (9.1 % and 7.8 %) and phase II study in Japan (11.1% and 11.1%) [5, 6]. The rates of grade 3 or 4 non-hematological toxicities such as diarrhea, anorexia, nausea, vomiting and fatigue in this study tended to be lower in comparison with those in the two previous studies [5, 6]. The lower incidence of non-hematological toxicities, which are directly linked to the patients’ symptoms, might be associated with the lower discontinuation rate (12.9%) from adverse events in our study. However, the rate of peripheral sensory neuropathy (9.7%) tended to be higher in this study than in the French (9.0%) and Japanese studies (5.6%) [5, 6]. One possible reason for this finding might be that the total dose of oxaliplatin in our study might have been higher than in the other 2 studies, since the median number of treatment cycles in our study was 13, which was larger than that in the other studies.

In 2013, Von Hoff et al. reported that nab-paclitaxel plus GEM significantly improved the RR, OS, and PFS, compared to GEM in patients with metastatic pancreatic adenocarcinoma (the phase III MPACT study) [10]. However, there have been no clear evidence regarding which regimen, FX or nab-paclitaxel plus GEM, is more effective as a first-line treatment in patients with unresectable advanced pancreatic cancer. Considering the higher adverse event rates of FX, it might be difficult to perform FX as a second-line treatment, at which time the patient condition is usually not as good as that during the first-line treatment. In that respect, a treatment strategy with FX as first-line treatment followed by nab-paclitaxel plus GEM as a second-line might be recommended, although further evaluation is required.

The UGT1A1 gene is responsible for the metabolism of SN-38, which is an active metabolite of irinotecan, and variants of UGT1A1 have been reported to intensify myelosuppression, such as severe neutropenia [6]. A study by Takahara et al [11]. evaluating the associations of variants in the UGT1A1 gene with the safety and efficacy of irinotecan monotherapy for pancreatic cancer in a Japanese cohort showed higher rates of neutropenia and anorexia in patients with UGT1A1 ^*^6 and/or ^*^28. In our study, patients with homozygous UGT1A1^*^6 or UGT1A1^*^28, or heterozygous UGT1A1^*^6 and UGT1A1^*^28 were excluded, similar to in the Japanese phase II study of FX [6]. Considering insufficient data on the safety of the FX regimen in patients with homozygous UGT1A1^*^6 or UGT1A1^*^28, or with heterozygous UGT1A1^*^6 and UGT1A1^*^28, as well as the higher rates of neutropenia-related adverse events in our study evaluating mFX, further modification of the irinotecan dose and intensive observation of the clinical course should be considered in patients with homozygous UGT1A1^*^6 or UGT1A1^*^28, or heterozygous UGT1A1^*^6 and UGT1A1^*^28, especially in Japanese patients.

This study had several limitations. First, both locally advanced and metastatic pancreatic cancers were enrolled in this study as unresectable advanced pancreatic cancer. Because of that, the OS might have tended to be better than that in the previous studies which included only metastatic pancreatic cancer, although the results of the subanalysis of metastatic cases still showed comparable efficacy with the FX phase II/III study in France [5] and phase II study in Japan [6]. Second, in our study, patients with homozygous UGT1A1^*^6 or UGT1A1^*^28, or heterozygous UGT1A1^*^6 and UGT1A1^*^28 were excluded, similar to in the Japanese phase II study of FX [6]; this might have affected the rates of adverse events, as compared with those in other studies without exclusion criteria related to the UGT1A1 status. Finally, since there was no comparing arm in this study, further comparison studies between mFX and other regimens including regular FX are required to confirm the efficacy and safety of mFX. On the other hand, the major strength of this study was its prospective multi-center study design.

In conclusion, this open-labeled phase II study showed that the RR, PFS, and OS of mFX in Japanese patients with unresectable advanced pancreatic cancer including both locally advanced and metastatic cancer were comparable to those of full-dose FX, and the rates of non-hematological adverse events were also well-controlled. However, the rates of hematological adverse events, especially neutropenia and FN, were not improved by initial dose reduction. Careful observation of the patients, especially during the first treatment cycle and in those with a biliary stent, is required to prevent the development of severe hematological adverse events. Further comparison studies are required to confirm the efficacy and safety of mFX.

## PATIENTS AND METHODS

### Study design

This study was designed as an open-label, multicenter prospective phase II study of first-line mFX for unresectable advanced pancreatic cancer. The primary endpoint was the RR. The secondary endpoints were OS, PFS, and the frequency and degree of adverse events. All patients provided written informed consent. The study was carried out in accordance with the Declaration of Helsinki. The study protocol was approved by the institutional review board of each participating institution (Gifu University Hospital, 26-200; Gifu Municipal Hospital, 2014-222; Gifu Prefectural General Medical Center, 2014-171) and was registered at the UMIN Clinical Trials Registry (UMIN000015376).

### Patient eligibility criteria

Patients were eligible if they had histologically proven adenocarcinoma or adenosquamous carcinoma of the pancreas; unresectable advanced pancreatic cancer, including locally advanced and metastatic cancer; an Eastern Cooperative Oncology Group performance status of 0 or 1; and age of 20-75 years. In this study, locally advanced pancreatic cancer was defined as either i) involvement of the celiac, supra mesenteric, common hepatic, or proper hepatic arteries, or ii) involvement of the portal vein or supra mesenteric vein which was considered as difficult for reconstruction, without clear evidence of distant metastasis based on enhanced computed tomography within 4 weeks of protocol entry. Further, to fulfill the eligibility criteria, patients were required to have an absolute neutrophil count >2000/mm^3^, platelet count >100,000/mm^3^, hemoglobin >9.0 g/dL, aspartate transaminase and alanine transaminase <150 U/L, creatinine <1.2 mg/dL, creatinine clearance >50 mL/min, possible oral intake, and no electrocardiographic abnormalities within 4 weeks of protocol entry. The patients were excluded from the study if they met any of the following conditions: pulmonary fibrosis or interstitial pneumonia; watery stools; active infection; serious concomitant diseases; apparent pleural effusion or ascites; history of prior chemotherapy or radiation therapy; UGT genetic polymorphisms (homozygous UGT1A1^*^6 or UGT1A1^*^28, or heterozygous UGT1A1^*^6 and UGT1A1^*^28); metastasis to the central nervous system; synchronous double cancer or metachronous double cancer with a disease-free period <3 years; patients using atazanavir, flucytosine, phenytoin, or warfarin potassium; patients who were pregnant, lactating, or planning a pregnancy; those with serious mental disorders; and anyone considered ineligible by the investigators.

### Treatment

Patients received mFX, consisting of oxaliplatin intravenously at 85 mg/m^2^ for 2 hours, leucovorin intravenously at 400 mg/m^2^ for 2 hours, irinotecan intravenously at 150 mg/m^2^ for 90 minutes, and 5-FU intravenously at 2400 mg/m^2^ over 46 hours. To prevent nausea and vomiting, aprepitant 125 mg was administered orally, and dexamethasone 6.6 mg and granisetron hydrochloride 3 mg were administered intravenously on day 1, followed by aprepitant 80 mg and dexamethasone 8 mg administered orally on days 2 and 3. The treatment was continued in repeating 14-day cycles as long as the regimen was tolerated and/or until disease progression, discontinuation decided by the investigators, or patient refusal. For careful safety evaluation, all patients were admitted to the hospital until the administration of the second round of mFX. Granulocyte-colony stimulating factor (G-CSF) was not administered as prophylaxis against neutropenia or FN.

The treatment was delayed in case of one or more of the following: absolute neutrophil count <1500/mm^3^, platelet count <75,000/mm^3^, total bilirubin >3.0 mg/dL, aspartate transaminase and alanine transaminase >150 U/L, creatinine >1.5 mg/dL, FN, grade ≥3 peripheral sensory neuropathy, and grade ≥3 diarrhea. The treatment was resumed upon recovery from these criteria. The dose of oxaliplatin and irinotecan were reduced beased on the following criteria: delayed cycle due to absolute neutrophil count (ANC) <1500mm^3^, platelet count <50000mm^3^, total bilirubin >2.0mg/dL, grade 2 peripheral sensory neuropathy, FN, and diarrhea with fever. The dose of oxaliplatin was reduced before that of irinotecan. The dose of 5-FU was reduced in the following cases: grade 3 or higher diarrhea, mucositis and palmar-plantar erythrodysesthesia syndrome. The dose reduction of oxaliplatin, irinotecan, and 5-FU were set to 65mg/m^2^ and 50mg/m^2^, 120mg/m^2^, and 1800mg/m^2^ and 1200mg/m^2^, respectively. If grade 3 or higher peripheral sensory neuropathy occurred, oxaliplatin was omitted until recovery to grade 2 or lower peripheral sensory neuropathy; however, mFX could be continued without oxaliplatin. The dosage adjustment of mFX followed that in the above mentioned Japanese phase II study of FX [6], except for the difference in the initial dose. The treatment also could be delayed by the attending physician based on general condition or convenience of patients.

### Assessment

Throughout the whole treatment course, the patients were assessed for their general condition and any possible adverse events by physical and blood examinations including complete blood count and blood chemical tests, generally once a week by the attending physicians. The carbohydrate antigen 19-9 levels were monitored at baseline and every 4 weeks. The treatment response was assessed by the radiologists at each center by comparing the computed tomography scans taken at baseline to scans taken at least every 12 weeks after treatment initiation. Adverse events were scored by using the National Cancer Institute Common Terminology Criteria for Adverse Events version 4.0. The radiologic tumor response was evaluated using the Response Evaluation Criteria in Solid Tumors version 1.0.

### Statistical analysis

The RRs were defined as the best accomplished response rates. OS was calculated from the date of initiation of mFX to the date of death. PFS was calculated from the date of initiation of mFX to the date of disease progression. The relative dose intensity was calculated as the ratio of the amount of drug actually administered to the amount of the standard regimen during the whole treatment period from the date of initiation to completion of mFX. The outcomes of OS, PFS, and RR were calculated with the corresponding 95% confidence interval (CI). OS and PFS were estimated using the Kaplan-Meier method. All statistical analyses were performed using JMP 10.0 (SAS Institute, Inc, Cary, NC, USA).

The RRs associated with GEM and FX were reported as 9.4% and 31.6%, respectively in the phase II/III study of FX in France [5]. In the phase II study of FX in Japan, the RR associated with FX was reported as 38.9% [6]. From the above results, the threshold RR and the expected RR were set at 9% and 35%, respectively. Subsequently, the sample size was calculated as 25 patients by one-arm binomial sample size calculation, with a power of at least 90% and a one-sided significance level of 2.5%. Accordingly, the target sample size was set as 28 patients to account for an omission rate of 10%.

## Abbreviations

FX: FOLFIRINOX
AE: adverse event
mFX: modified FOLFIRINOX
GEM: gemcitabine
5-FU: 5-fluorouracil
OS: overall survival
PFS: progression free survival
RR: response rate
FN: febrile neutropenia
G-CSF: granulocyte-colony stimulating factor
ANC: absolute neutrophil count
CI: confidence interval
UGT1A1: uridine diphosphate-glucuronosyltransferase 1A1
